# Subsequent surgical treatment or maintenance immunotherapy in stage III lung cancer patients achieving a favorable response following neoadjuvant immunotherapy: A matched retrospective cohort study from the surgical perspective

**DOI:** 10.1111/1759-7714.15247

**Published:** 2024-02-27

**Authors:** Fuqiang Dai, Cong Chen, Guanyu Zhou, Xintian Wang, Longyong Mei, Nanzhi Luo, Wenjing Zhou, Tao Li, Bo Deng, Lunxu Liu, Yun Wang

**Affiliations:** ^1^ Department of Thoracic Surgery and Institute of Thoracic Oncology Frontiers Science Center for Disease‐related Molecular Network, West China Hospital of Sichuan University Chengdu China; ^2^ Department of Thoracic Surgery Daping Hospital, Army Medical University Chongqing China; ^3^ Laboratory of Mitochondria and Metabolism, National‐Local Joint Engineering Research Centre of Translational Medicine of Anesthesiology West China Hospital, Sichuan University Chengdu China

**Keywords:** advanced stage III non‐small cell lung cancer, immunotherapy and chemotherapy, promising prognosis, surgery intervention

## Abstract

**Background:**

Current treatment strategies for advanced non‐small cell lung cancer (NSCLC) are highly individualized and subject to ongoing debates. In the era of immunotherapy, surgery assumes a critical role. The aim of this study was to investigate if subsequent surgical intervention, following a favorable response to immunotherapy and chemotherapy, could yield a more favorable prognosis for patients with advanced stage III NSCLC compared to the continuation of immunotherapy and chemotherapy.

**Methods:**

We included patients whose tumors exhibited a favorable response (including partial response [PR] and complete response [CR]) to immunotherapy and chemotherapy. These patients were categorized into two groups based on their subsequent treatment plans: surgical and nonsurgical (continuation of maintenance immunotherapy and chemotherapy). The efficacy and long‐term prognosis of these groups were compared after matching them in a 1:1 ratio using propensity scores.

**Results:**

In total, 186 patients (93 in each group) were included in this study after matching via propensity scores. The 1‐ and 3‐year overall survival (OS) and progression‐free survival (PFS) rates were 96.0%, 88.5%, and 93.1%, 80.7% in the surgical group, and 93.2%, 83.1%, and 57.7%, 50.4% in the nonsurgical group, respectively. Patients in the surgical group exhibited significantly superior PFS and OS compared to those in the nonsurgical group (*p* = 0.025 and *p* = 0.00086). Univariate and multivariate analyses confirmed ΔBMI, Δtumor size reduction, tumor response, earlier clinical stage (IIIb vs. IIIa), and surgery as independent protective factor for patient prognosis. We further selected 101 patients with CR (39 in the surgical group and 62 in the nonsurgical group) and found that patients in the surgical group were significantly better in both PFS and OS. Our subgroup analysis in postoperative patients demonstrated that different surgical strategies did not significantly affect the long‐term prognosis of patients (PFS and OS) but could impact their perioperative experience.

**Conclusion:**

Patients with advanced stage III NSCLC, whose tumors achieved PR and CR after 2–4 cycles of immunotherapy combined with chemotherapy, experience a more promising prognosis with subsequent surgical intervention compared with the continued immunotherapy. Despite encountering formidable obstacles, such as protracted surgical procedures and associated trauma, we must rise to the challenge and unleash the power of surgery after immunotherapy in advanced NSCLC.

## Graphical abstract

In the era of immunotherapy, surgery presents a promising avenue for the potential cure of non‐small cell lung cancer (NSCLC). Various surgical approaches like minimal invasive surgery and bronchial or vascular sleeve resection, frequently aim to attain R0 resection without significant difference in survival rates. **Perspective Statement:** The notion of curing cancer patients with medication remains an elusive dream. Despite facing formidable obstacles after neoadjuvant therapy, we must rise to the challenge and unleash the power of surgery for curing cancer.

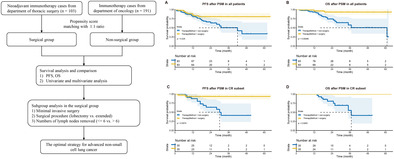

## INTRODUCTION

Lung cancer is a prevalent and fatal malignancy globally.^1^ Advances in antitumor treatments have led to various therapeutic approaches based on lung cancer stages, significantly enhancing patient survival. Surgical interventions offer a favorable prognosis for early‐stage lung cancer patients.^2^ Individuals diagnosed with advanced or metastatic lung cancer can benefit from radiotherapy, chemotherapy, targeted therapy, and immunotherapy, all with well‐defined indications.^3^ Nevertheless, the treatment strategy for advanced lung cancer remains contentious due to the complexity and heterogeneity of tumors.^4^

Recent studies have suggested that neoadjuvant immunotherapy, either alone or in combination with chemotherapy, significantly improves the prognosis of patients with middle to advanced stage resectable lung cancer.^5^, ^6^, ^7^ In cases of unresectable advanced lung cancer, the primary treatment strategies involve concurrent or sequential radiotherapy and immunochemotherapy.^2^, ^8^ Despite the recognition of AJCC guidelines in clinical practice, the determination of resectability for advanced lung cancer, especially at stage III, often relies on the surgeon's expertise, surgical skills, and the healthcare facility's medical proficiency. Additionally, deciding on surgical resection for patients initially diagnosed with unresectable lung cancer after a positive response to immunotherapy and chemotherapy remains a subject of debate, influenced by the surgeon's experience.^9^ Notably, patients exhibiting tumor regression following treatment with immune checkpoint inhibitors (ICIs) tend to experience pronounced tissue adhesions and fibrosis due to tumor necrosis and subsequent tissue repair. This phenomenon complicates surgical interventions and significantly increases postoperative complications. Bott et al. documented a notable 32% incidence of postoperative complications among a cohort of 19 patients initially diagnosed with inoperable lung cancer, who subsequently underwent lobectomy after ICI treatment.^10^ In such circumstances, less experienced surgeons often opt a conservative approach, which includes ongoing maintenance immunotherapy, among other treatment modalities. Examining whether surgical intervention provides a superior prognosis compared to maintenance immunotherapy and chemotherapy for patients in this subgroup, manifesting tumor response and potential surgical eligibility post‐immunotherapy, merits investigation.

To address this query, this study retrospectively gathered clinical data from patients with non‐small cell lung cancer (NSCLC) at an advanced stage III, a cohort for which treatment regimens are highly debated. All enrolled patients exhibited a positive response of their tumors to immunotherapy and chemotherapy. We compared their prognoses based on whether they underwent subsequent surgical interventions. By scrutinizing post‐immunotherapy treatment options for patients experiencing tumor response, the aim of this study was to serve as a vital and credible reference in formulating surgical intervention protocols during antitumor therapy for individuals with advanced stage III NSCLC.

## METHODS

### Patient selection

The study received approval from the hospital's ethics review board (Ethics Committee of Army Medical Center of PLA, approval no.: 2021‐273) on December 10, 2021. Given the retrospective nature of this analysis, individual consent was not required. The study was performed in accordance with the Declaration of Helsinki. This retrospective study included patients diagnosed with stage III NSCLC, who received neoadjuvant immunotherapy and chemotherapy followed by either surgical intervention or continued maintenance immunotherapy and chemotherapy from January 1, 2018, to May 31, 2022, at Daping Hospital, Army Medical University. Lung cancer samples for pathological diagnosis were obtained through fiberoptic bronchoscopy or percutaneous pulmonary puncture. Staging of the tumor was determined using positron emission tomography‐computed tomography (PET‐CT) and chest computed tomography (CT) according to the eighth edition AJCC TNM Lung Cancer Stage Classification.

Inclusion criteria were as follows: (1) Age between 18 and 75 years. (2) Completion of 2–4 cycles of neoadjuvant immunotherapy combined with chemotherapy in the surgical group (selected from patients receiving neoadjuvant immunotherapy and chemotherapy in the department of thoracic surgery) and completion of four cycles of immunotherapy combined with chemotherapy in the nonsurgical group (selected from patients receiving immunotherapy and chemotherapy in the department of oncology). (3) Tumors in both groups exhibiting a favorable response (including partial response [PR] and complete response [CR]). (4) Having detailed postoperative pathological results, including primary tumor and lymph nodes. (5) Eastern Cooperative Oncology Group (ECOG) performance status score of 0–2. The exclusion criteria were as follows: (1) Poor cardiopulmonary function leading to inability to tolerate surgery. (2) Presence of other malignant tumors or distant metastases. (3) Positivity for EGFR or ALK rearrangement/fusion mutations.

### Study design and endpoints

In this study, eligible patients were categorized into two groups: the surgical group comprised patients who underwent surgical treatment after their tumors achieving a favorable response following neoadjuvant immunotherapy, whereas the nonsurgical group consisted of patients who continued immunotherapy.

**FIGURE 1:**
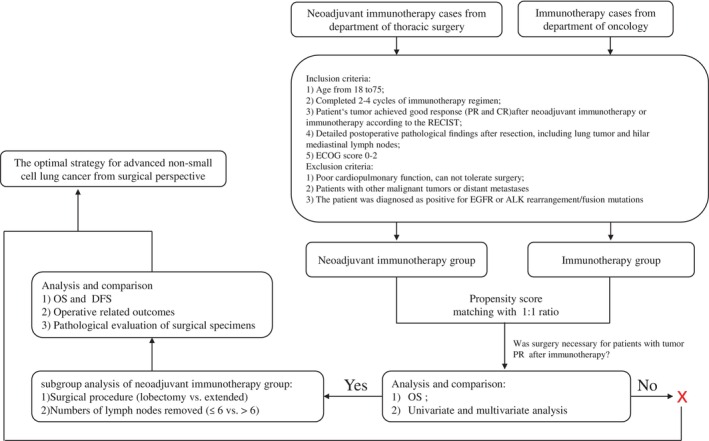
Flow chart summarizing the process of enrollment and prognostic analysis of surgical and nonsurgical groups after immunotherapy in patients with advanced stage III non‐small cell lung cancer. PFS, progression‐free survival; OS, overall survival; PR, partial response; CR, complete response; RECIST, response evaluation criteria in solid tumors; ECOG, Eastern Cooperative Oncology Group.

As depicted in Figure 1, we initially identified patients with tumor efficacy achieving a favorable response after neoadjuvant immunotherapy in the thoracic surgery department and gathered their baseline data, including age, gender, and lung cancer type. Simultaneously, three experienced thoracic surgeons with over 10 years of clinical experience evaluated whether patients were suitable for surgery based on follow‐up CT and fiberoptic bronchoscopy results. Patients were included in the nonsurgical group only when at least two or more surgeons concluded that surgery was indicated. Subsequently, we used propensity score matching based on the baseline data of patients in the two group in a 1:1 ratio and compared prognoses between the two groups.

The endpoints of the study were overall survival (OS) and progression‐free survival (PFS). OS was defined as the duration from the commencement of treatment to the patient's date of death or last follow‐up. For patients in the surgical group, PFS was defined as the interval between the surgical procedure and either tumor recurrence or mortality, and for patients in the nonsurgical group, PFS was specified as the period from the point a patient was evaluated as achieving a favorable response to disease progression.

### Treatment protocols

Both groups received a combination of chemotherapy along with immunotherapy. Chemotherapy regimens comprised pemetrexed and nedaplatin for adenocarcinoma and paclitaxel liposome/docetaxel and nedaplatin for squamous carcinoma. The immunotherapy protocols featured PD‐1 inhibitors, including tislelizumab, nivolumab and pembrolizumab. Patients underwent 2–4 cycles of therapy, followed by a chest‐enhanced CT and PET‐CT 3–6 weeks later to assess tumor changes and therapeutic effects.

In case of patients with a potential indication for surgery, surgical resection was performed in adherence to the Guidelines for the Diagnosis and Treatment of Primary Lung Cancer, which included pneumonectomy, lobectomy, and bronchial or vascular sleeve resection. Post‐surgery, patients received 4–6 cycles of adjuvant therapy with the same regimen as before. In case of tumor recurrence during follow‐up, the patient's tolerance to current treatment and tumor status were assessed through comprehensive examination. Pathological and genetic testing of the recurrent lesion was conducted, and an individualized treatment plan was developed and implemented following evaluation by a multidisciplinary team (MDT) and obtaining informed consent from the patient.

In the nonsurgical group, PD1 inhibitors and chemotherapy were used based on the condition of the patient, and imaging tests were performed after four cycles of therapy to evaluate tumor changes. Maintenance of the current immunotherapy regimen and regular follow‐up were conducted for patients. In case of tumor progression, recurrence, or metastasis during the follow‐up period, a comprehensive multidisciplinary assessment by MDT is still required to formulate a personalized adjuvant treatment strategy.

### Evaluation of pathological samples

Pathological staging and the percentage of residual tumor cells in surgically resected specimens after neoadjuvant immunotherapy in lung cancer patients were independently scored by two trained pathologists. Pathological complete response (pCR) was determined when no tumor cells were detected in all sections of the patient's pathology samples after neoadjuvant therapy. In cases of disagreement between pathologists, a consensus was reached through joint re‐review and discussion using a multihead microscope.

### Propensity score matching and statistical analysis

A multivariate logistic regression model was used to establish propensity scores based on clinical baseline data of patients, including age, gender, smoking status, forced expiratory volume in the first second (FEV1%), body mass index (BMI), prognostic nutritional index (PNI), tumor size, tumor location, pathological tumor type, clinical stage, PD‐L1, CR/PR, and tumor type. Patients from the surgical group were then matched with patients from the nonsurgical group in a 1:1 ratio using the nearest neighbor matching algorithm, with a common caliper value of 0.1.

Data analysis was performed using SPSS 26.0 software (IBM SPSS Statistics, RRID:SCR_019096). Continuous variable data conforming to a normal distribution are presented as mean ± standard deviation (±s). The two‐tailed *t*‐test or rank sum test was utilized for group comparisons. Non‐normally distributed data are described using the median and upper and lower quartiles [M (P25, P75)], and the Mann–Whitney U test was employed for intergroup comparisons. Categorical variables are reported in terms of frequency and percentage (%), with comparisons between groups carried out using *χ* ^2^ or Fisher's exact test. Survival curves were generated using the Kaplan–Meier method, and differences between survival curves were assessed using the log‐rank test. Univariate and multivariate analyses were performed using the backward stepwise Cox proportional hazards model to identify independent prognostic factors. Variables with a *p* ≤ 0.10 in the univariate analysis were included in the multivariate Cox proportional hazards model. A significance level of *p* < 0.05 was applied in this study.

## RESULTS

### Patient clinical characteristics

A total of 103 patients in the surgical group and 191 patients in the nonsurgical group were initially included, all diagnosed with advanced stage III lung cancer (covering IIIa, IIIb, and IIIc). Parameters such as gender, BMI before immunotherapy, smoking status, alcohol use, FEV1%, tumor size before immunotherapy, tumor location, tumor type, expression of PD‐L1, CR/PR and clinical stage before immunotherapy remained consistent between the two groups. The majority of enrolled patients were male (90.6% and 93.7%), smokers (86.5% and 84.3%), alcohol drinkers (81.2% and 83.8%), and had squamous carcinoma (75.0% and 76.4%). After propensity score matching, a total of 186 patients (93 patients in each group) were finally included, and there were no significant differences in baseline characteristics between the two groups (Table 1).

**TABLE 1:** Demographic, clinical, and pathological factors in surgical and nonsurgical groups.

Characteristics	Unmatched patients			Matched patients		
	Surgical	Nonsurgical	*p*‐value	Surgical	Nonsurgical	*p*‐value
**Patients (*n*)**	103	191		93	93	
**Age (years)**	59.53 ± 7.75	63.76 ± 8.32	<0.001	59.83 ± 7.77	61.81 ± 8.61	0.102
**Gender, *n* (%)**			0.180			0.620
Male	92 (89.3%)	179 (93.7%)		83 (89.2%)	85 (91.4%)	
Female	11 (10.7%)	12 (6.3%)		10 (10.8%)	8 (8.6%)	
**BMI (kg/m** ^**2**^ **)**						
Pre‐nICT	23.19 ± 2.88	22.57 ± 2.92	0.082	23.14 ± 2.88	22.79 ± 3.04	0.421
Post‐nICT	23.86 ± 2.82	22.84 ± 2.97	0.005	23.78 ± 2.77	23.25 ± 2.91	0.212
ΔBMI^a^	0.70 (0.10, 1.60)	0.20 (−0.40, 0.90)	0.001	0.70 (0, 1.60)	0.40 (−0.40, 1.10)	0.156
**PNI**	46.99 ± 5.35	44.13 ± 6.06	<0.001	46.51 ± 5.32	45.28 ± 6.69	0.115
**CCI**	2 (1, 3)	2 (2, 3)	0.033	2 (1, 3)	2 (1, 3)	0.459
**Smoking status**			0.969			0.840
Absent	16 (15.5%)	30 (15.7%)		14 (15.1%)	15 (16.1%)	
Present	87 (84.5%)	161 (84.3%)		79 (84.9%)	78 (83.9%)	
**Alcohol use**			0.373			0.704
No	21 (20.4%)	31 (16.2%)		18 (19.4%)	16 (17.2%)	
Yes	82 (79.6%)	160 (83.8%)		75 (80.6%)	77 (82.8%)	
**FEV1%**	87.00 (79.00, 99.60)	85.20 (77.80, 96.50)	0.269	87.00 (77.85, 98.50)	84.00 (75.30, 92.40)	0.225
**Tumor size (cm)** ^‡^						
Pre‐nICT	5.0 (3.7, 6.4)	5.3 (3.9, 6.7)	0.391	5.0 (3.8, 6.3)	4.9 (3.9, 6.4)	0.983
Post‐nICT	2.7 (1.7, 2.8)	3.5 (2.4, 5.2)	<0.001	2.7 (1.8, 2.9)	2.8 (1.9, 3.7)	0.063
Δtumor size	2.4 (1.5, 3.9)	1.5 (0.5, 2.6)	<0.001	2.3 (1.4, 3.7)	2.2 (1.1, 2.8)	0.094
**Tumor response**			0.352			0.458
PR	64 (62.1%)	129 (67.5%)		56 (60.2%)	51 (54.8%)	
CR	39 (37.9%)	62 (32.5%)		37 (39.8%)	42 (45.2%)	
**Tumor location**			0.136			0.083
RUL	34 (33.0%)	67 (35.1%)		31 (33.3%)	36 (38.7%)	
RML	8 (7.8%)	5 (2.6%)		6 (6.5%)	2 (2.2%)	
RLL	17 (16.5%)	33 (17.3%)		16 (17.2%)	11 (11.8%)	
LUL	23 (22.3%)	58 (30.4%)		23 (24.7%)	35 (37.6%)	
LLL	21 (20.4%)	28 (14.7%)		17 (18.3%)	9 (9.7%)	
**Tumor type**			0.510			0.363
Squamous cell	77 (74.8%)	146 (76.4%)		69 (74.2%)	70 (75.3%)	
Adenocarcinoma	24 (23.3%)	44 (23.0%)		22 (23.7%)	23 (24.7%)	
Others	2 (1.9%)	1 (0.5%)		2 (2.1%)	0 (0%)	
**Clinical stage** ^b^			0.008			0.229
IIIa	64 (62.1%)	83 (43.5%)		56 (60.2%)	45 (48.4%)	
IIIb	31 (30.1%)	91 (47.6%)		29 (31.2%)	40 (43.0%)	
IIIc	8 (7.8%)	17 (8.9%)		8 (8.6%)	8 (8.6%)	
**PD‐L1**			0.755			0.304
Negative	44 (42.7%)	78 (40.8%)		45 (48.4%)	52 (55.9%)	
Positive	59 (57.3%)	113 (59.2%)		48 (51.6%)	41 (44.1%)	
**Immunotherapy**			0.514			0.141
Nivolumab	23 (22.3%)	32 (16.8%)		24 (25.8%)	14 (15.1%)	
Pembrolizumab	30 (29.1%)	54 (28.3%)		27 (29.0%)	26 (28.0%)	
Tislelizumab	46 (44.7%)	92 (48.2%)		42 (45.2%)	53 (57.0%)	
Others	4 (3.9%)	13 (6.8%)				

*Note*: In the surgical group before matching, there was one patient with R1 resection and six patients with R2 resection; Matching with a 1:1 propensity score was used with a caliper value set = 0.1, and 93 pairs of samples were eventually matched.

Abbreviations: CCI, Charlson comorbidity index; CR, complete response; LLL, left lower lung; LUL, left upper lung; nICT, Neoadjuvant immunotherapy combined with chemotherapy; PNI, prognostic nutritional index; PR, partial response; RLL, right lower lung; RML, right middle lung; RUL, right upper lung; Tumor response, tumor response to therapy; Δ, change in values before and after neoadjuvant immunotherapy combined with chemotherapy.

^a^ Since ΔBMI values were not normally distributed, we used the non‐parametric rank sum test.

^b^ Clinical stage prior to immunotherapy.

### Univariate and multivariate survival analysis between surgical and nonsurgical groups

**FIGURE 2:**
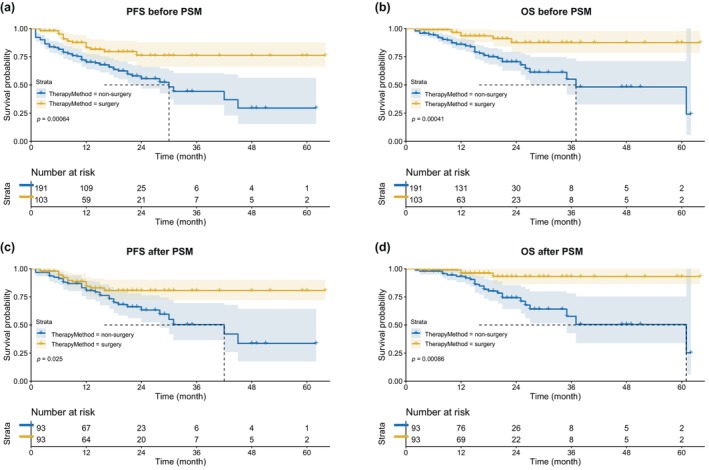
Kaplan–Meier curves for survival before and after propensity score matching between surgical and nonsurgical groups. (a) PFS between surgical and nonsurgical groups before PSM. (b) OS between surgical and nonsurgical groups before PSM. (c) PFS between surgical and nonsurgical groups after PSM. (d) OS between surgical and nonsurgical groups after PSM. PFS, progression‐free survival; OS, overall survival; PSM, propensity score matching.

In our study, we conducted a follow‐up spanning 6 to 68 months and analyzed overall survival (OS) and progression‐free survival (PFS) (as shown in Figure 2). There were nine deaths in the surgical group and 47 deaths in the nonsurgical group. Regarding tumor progression, 18 patients in the surgical group experienced tumor recurrence after surgery, while 71 patients in the nonsurgical group experienced tumor progression. In the surgical group, the 1‐ and 3‐year OS and PFS rates were 96.5%, 87.6%, and 87.5%, 76.2%, respectively. In the nonsurgical group, the rates were 86.1%, 72.2%, and 55.1%, 44.3%, respectively. Significant differences were observed in PFS and OS between the two groups (*p* = 0.00064 and *p* = 0.00041, Figure 2a, b).

Following 1:1 propensity score matching, patients in the surgical group also exhibited significantly better PFS and OS than patients in the nonsurgical group (*p* = 0.025 and *p* = 0.00086, Figure 2c,d). The 1‐ and 3‐year OS and PFS rates were 96.0%, 88.5%, and 93.1%, 80.7% in the surgical group and 93.2%, 83.1%, and 57.7%, 50.4%, respectively, in the nonsurgical group.

Additionally, we conducted a univariate analysis of clinical indicators such as age, gender, BMI, PNI, CCI, smoking status, alcohol use, FEV1%, and whether to have surgery, among all patients after matching (as shown in Table 2). The results showed that ΔBMI (*p* = 0.039), Δtumor size reduction (*p* = 0.085), tumor response (*p* = 0.029) Clinical stage (IIIb vs. IIIa) (*p* = 0.006), and whether to have surgery (*p* = 0.003) were potential factors affecting the prognosis of patients. Furthermore, the multivariate COX regression analysis showed that tumor size reduction (HR: 0.358, 95% CI: 0.149–0.862, *p* = 0.022) and surgery (HR: 0.280, 95% CI: 0.093–0.844, *p* = 0.024) were protective factors for the prognosis of patients with advanced lung cancer, and clinical stage was a risk factor (HR: 2.959, 95% CI: 1.194–7.332, *p* = 0.019).

**TABLE 2:** Univariate and multivariate analysis of overall survival for advanced stage III non‐small lung cancer in patients with tumor good response after immunotherapy.

Variables	Univariate analysis*					Multivariate analysis				
	B	SE	Wals	OR (95% CI)	*p*‐value*	B	SE	Wals	OR (95% CI)	*p*‐value
Age (≥60 vs. <60 years)	0.467	0.396	1.395	1.596 (0.735–3.467)	0.238					
Gender (female vs. male)	−0.050	0.551	0.008	0.951 (0.323–2.799)	0.927					
BMI pre‐nICT (≥22 vs. <22 kg/m^2^)	−0.325	0.387	0.707	0.722 (0.339–1.541)	0.400					
BMI post‐nICT (≥22 vs. <22 kg/m^2^)	−0.221	0.390	0.322	0.801 (0.373–1.722)	0.571					
ΔBMI (increase vs. decrease)	−0.795	0.385	4.257	0.452 (0.212–0.961)	**0.039**	−0.556	0.397	1.962	0.573 (0.263–1.249)	0.161
PNI (>45 vs. ≤45)	−0.295	0.388	0.577	0.744 (0.348–1.594)	0.447					
CCI (>2 vs. ≤2)	0.401	0.383	1.100	1.494 (0.706–3.161)	0.294					
Smoking status (present vs. absent)	−0.026	0.468	0.003	0.974 (0.389–2.436)	0.955					
Alcohol use (yes vs. no)	0.354	0.497	0.507	1.425 (0.538–3.776)	0.477					
FEV1% (≥80% vs. <80%)	−0.216	0.396	0.298	0.805 (0.370–1.751)	0.585					
Tumor size post‐nICT (>3 vs. ≤3 cm)	0.535	0.389	1.891	1.708 (0.796–3.661)	0.169					
Δ tumor size reduction (>2.5 vs. ≤2.5 cm)	−0.710	0.412	2.967	0.492 (0.219–1.103)	**0.085**	−1.027	0.448	5.245	0.358 (0.149–0.862)	**0.022**
Tumor response (CR vs. PR)	−0.916	0.420	4.748	0.400 (0.176–0.912)	**0.029**					
Tumor location (right vs. left lobe)	−0.128	0.123	1.082	0.880 (0.692–1.119)	0.298					
Tumor type (adenocarcinoma vs. squamous)	0.335	0.396	0.715	1.398 (0.643–3.041)	0.398					
PD‐L1 (positive vs. negative)	−0.041	0.381	0.011	0.960 (0.455–2.025)	0.915					
Immunotherapy	−0.030	0.249	0.015	0.970 (0.595–1.582)	0.903					
Clinical stage (IIIb vs. IIIa)	1.250	0.452	7.632	3.489 (1.438–8.466)	**0.006**	1.085	0.463	5.489	2.959 (1.194–7.332)	**0.019**
Clinical stage (IIIc vs. IIIa)	0.830	0.703	1.392	2.293 (0.578–9.101)	0.238	0.946	0.760	1.549	2.574 (0.581–11.414)	0.213
Whether to have surgery (yes vs. no)	−1.618	0.541	8.943	0.198 (0.069–0.573)	**0.003**	−1.274	0.564	5.111	0.280 (0.093–0.844)	**0.024**

*Note*: Bold values indicated less than 0.1 and may be significant values.

Abbreviations: BMI, body mass index; CCI, Charlson comorbidity index; CI, confidence interval; CR, complete response; nICT, neoadjuvant immunotherapy combined with chemotherapy; OR, odds ratio; PNI, prognostic nutritional index; PR, partial response; tumor response, tumor response to therapy; Δ, change in values before and after neoadjuvant immunotherapy combined with chemotherapy.

* Parameters with *p* < 0.1 were included in the multivariate analysis.

### Survival analysis in patients with complete response

**FIGURE 3:**
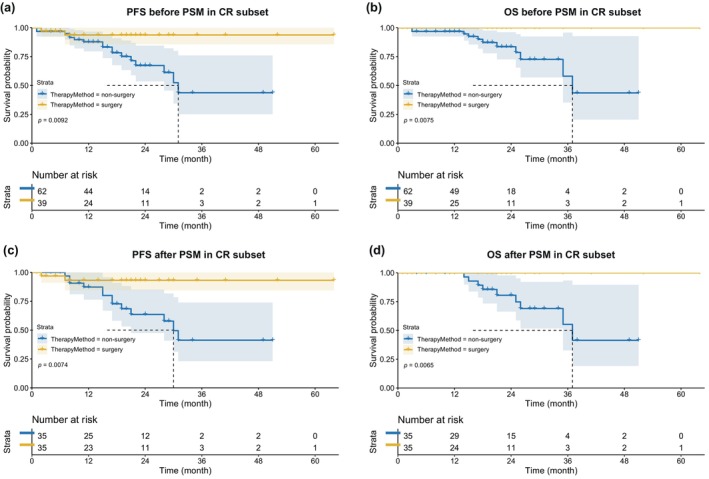
Kaplan–Meier curves for survival before and after propensity score matching between surgical and nonsurgical groups in the complete subset. (a) PFS between surgical and nonsurgical groups before PSM in CR subset. (b) OS between surgical and nonsurgical groups before PSM in CR subset. (c) PFS between surgical and nonsurgical groups after PSM in CR subset. (d) OS between surgical and nonsurgical groups after PSM in CR subset. PFS, progression‐free survival; OS, overall survival; PSM, propensity score matching; CR, complete response.

To further investigate the role of surgical intervention in patients with complete response, we selected patients with imaging complete response after 2–4 cycles of chemotherapy combined with immunotherapy for comparative analysis. After screening, 39 (surgical group) and 62 (nonsurgical group) patients were finally selected. Compared with the nonsurgical group, patients in the surgical group were younger (59.23 ± 8.07 vs. 63.37 ± 7.85, *p* = 0.012), and had a greater increase in BMI after neoadjuvant therapy (0.70 [0.20, 1.80] vs. 0.40 [−0.30, 1.00], *p* = 0.019). On other clinical parameters, there were no significant differences between the two groups (Table 3). Compared with the nonsurgical group, the surgical group was significantly better in both PFS and OS (Figure 3a,b).

**TABLE 3:** Comparative analyses for patients with complete response on imaging performance.

Characteristics	Unmatched patients			Matched patients		
	Surgical	Nonsurgical	*p*‐value	Surgical	Nonsurgical	*p*‐value
Patients (*n*)	39	62		35	35	
Age (years)	59.23 ± 8.07	63.37 ± 7.85	0.012	59.71 ± 8.21	61.66 ± 8.17	0.325
Gender, *n* (%)			0.639			1.000
Male	37 (94.9%)	60 (96.8%)		33 (94.3%)	33 (94.3%)	
Female	2 (5.1%)	2 (3.2%)		2 (5.7%)	2 (5.7%)	
BMI (kg/m^2^)						
Pre‐nICT	22.76 ± 2.57	22.59 ± 3.03	0.773	22.86 ± 2.62	22.56 ± 2.93	0.653
Post‐nICT	23.71 ± 2.59	22.94 ± 2.93	0.183	23.73 ± 2.55	23.22 ± 2.70	0.426
ΔBMI	0.70 (0.20, 1.80)	0.40 (−0.30, 1.00)	0.019	0.70 (0.20, 1.70)	0.50 (−0.30, 1.00)	0.323
PNI	46.58 ± 5.60	45.01 ± 6.08	0.196	46.67 ± 5.72	44.96 ± 6.38	0.242
CCI	2 (1, 3)	2 (1, 3)	0.093	2 (1, 3)	2 (1, 3)	0.648
Smoking status			0.946			1.000
Absent	3 (7.7%)	5 (8.1%)		3 (8.6%)	3 (8.6%)	
Present	36 (92.3%)	57 (91.9%)		32 (91.4%)	32 (91.4%)	
Alcohol use			0.125			0.324
No	8 (20.5%)	6 (9.7%)		7 (20.0%)	4 (11.4%)	
Yes	31 (79.5%)	56 (90.3%)		28 (80.0%)	31 (88.6%)	
FEV1%	86.00 (71.60, 100.00)	85.00 (78.48, 96.13)	0.638	86.40 (72.00, 102.00)	84.00 (75.40, 89.20)	0.442
Tumor size (cm)						
Pre‐nICT	4.8 (3.5, 6.2)	3.9 (3.3, 5.1)	0.053	4.8 (3.5, 5.6)	4.3 (3.5, 5.1)	0.350
Tumor location			0.294			0.348
RUL	11 (28.2%)	24 (38.7%)		11 (31.4%)	11 (31.4%)	
RML	2 (5.1%)	2 (3.2%)		1 (2.9%)	1 (2.9%)	
RLL	9 (23.1%)	9 (14.5%)		9 (25.7%)	4 (11.4%)	
LUL	9 (23.1%)	21 (33.9%)		8 (22.9%)	15 (42.9%)	
LLL	8 (20.5%)	6 (9.7%)		6 (17.1%)	4 (11.4%)	
Tumor type			0.141			0.201
Squamous cell	34 (87.2%)	48 (77.4%)		30 (85.7%)	26 (74.3%)	
Adenocarcinoma	4 (10.3%)	14 (22.6%)		4 (11.4%)	9 (25.7%)	
Others	1 (2.5%)	0		1 (2.9%)	0	
Clinical stage			0.258			0.580
IIIa	26 (66.7%)	31 (50.0%)		24 (68.6%)	20 (57.1%)	
IIIb	11 (28.2%)	26 (41.9%)		9 (25.7%)	13 (37.1%)	
IIIc	2 (5.1%)	5 (8.1%)		2 (5.7%)	2 (5.7%)	

Abbreviations: BMI, body mass index; CCI, Charlson comorbidity index; LLL, left lower lung; LUL, left upper lung; nICT, neoadjuvant immunotherapy combined with chemotherapy; PNI, prognostic nutritional index; RLL, right lower lung; RML, right middle lung; RUL, right upper lung; Δ, change in values before and after neoadjuvant immunotherapy combined with chemotherapy.

After propensity score matching, a total of 70 patients (35 patients in each group) were included, and there were no significant differences in baseline characteristics between the two groups (Table 3). Patients in the surgical group also exhibited significantly better PFS and OS than patients in the nonsurgical group (Figure 3c,d). This result suggests that even in tumors that are complete response on imaging, a surgical resection approach may kill the tumor more radically than drug therapy.

### Subgroup analysis of postoperative results in surgical group

Subsequently, we compared the impact of different surgical strategy on intraoperative parameters, postoperative short‐term, and long‐term prognosis in patients with advanced stage III NSCLC, including minimally invasive surgery and open thoracotomy groups, lobectomy and extended resection (larger area than lobectomy) groups, and less lymph node dissection (≤6) and more lymph node dissection (>6) groups.

#### Minimally invasive surgery or open thoracotomy

**FIGURE 4:**
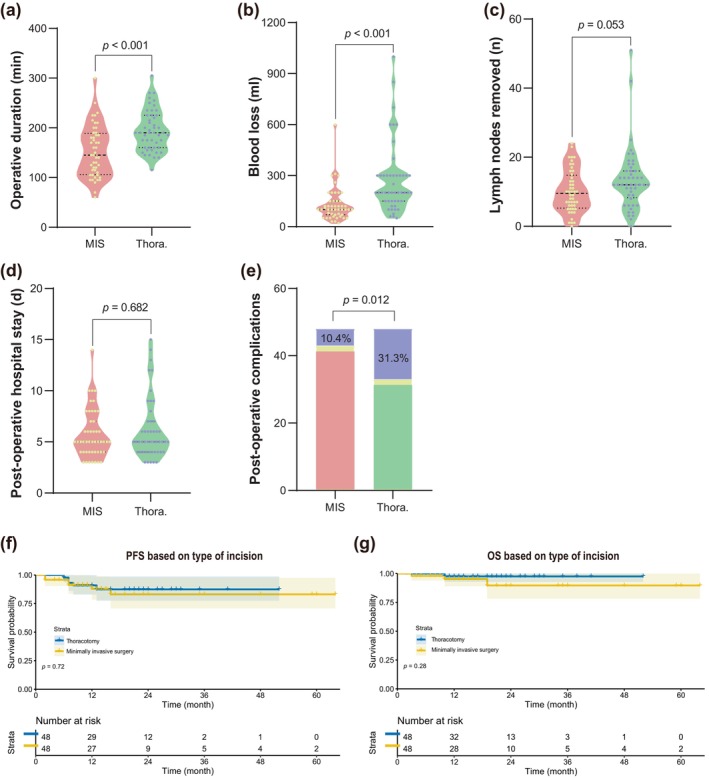
Comparisons of postoperative results between minimally invasive surgery and open thoracotomy group. (a) Operative duration (min). (b) intraoperative blood loss (mL). (c) Lymph nodes removed (n). (d) Postoperative hospital stay (days). (e) Rate of postoperative complications (%). (f) Kaplan–Meier curves for PFS. (g) Kaplan–Meier curves for OS. MIS, minimally invasive surgery; Thora., open thoracotomy; PFS, progression‐free survival; OS, overall survival.

As shown in Figure 4, the results suggested that open thoracotomy required a longer operation time (190 [160, 225] min vs. 145 [106, 189] min, *p* < 0.001, Figure 4a) and resulted in more blood loss (200 [150, 300] mL vs. 100 [70, 150] mL, *p* < 0.001, Figure 4b) than minimally invasive surgery. Open thoracotomy had a slightly advantageous number of lymph nodes removed (12 [8.25, 16] vs. 9.5 [5.25, 14.75], *p* = 0.053, Figure 4c), but patients also had a higher rate of postoperative complications (31.25% vs. 10.42%, *p* = 0.012 Figure 4e). Additionally, the length of postoperative hospitalization was relatively the same in both groups (5 [4, 7] days vs. 5 [4, 7] days, *p* = 0.682, Figure 4d). There was also no significant difference between the PFS and OS of patients in the open thoracotomy group compared with those in the MIS group (*p* = 0.72 and 0.28, respectively, Figure 4f,g). Taken together, MIS was proved to be a judicious decision for advanced stages III NSCLC to the extent that technology allowed.

#### Lobectomy or extended lobectomy

**FIGURE 5:**
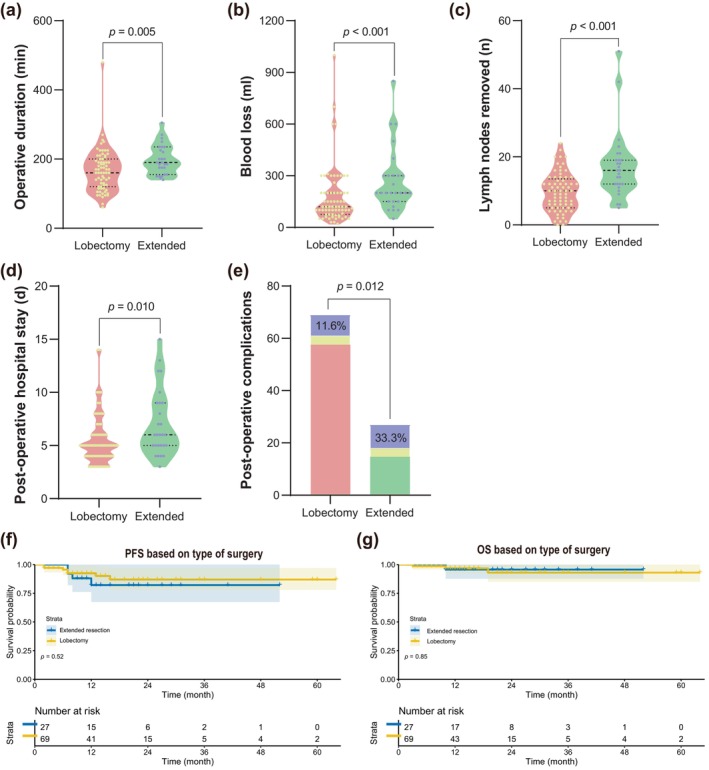
Comparison of postoperative results between lobectomy and extended lobectomy. (a) Operative duration (min). (b) Intraoperative blood loss (mL). (c) Lymph nodes removed (*n*). (d) Postoperative hospital stay (days). (e) Rate of postoperative complications (%). (f) Kaplan–Meier curves for PFS. (g) Kaplan–Meier curves for OS. PFS, progression‐free survival; OS, overall survival.

Extended surgical resection took more operative time (190 [155, 235] min vs. 160 [120, 200] min, *p* = 0.005, Figure 5a), and more intraoperative bleeding (200 [150, 300] mL vs. 120 [75, 200] mL, *p* < 0.001, Figure 5b) than lobectomy. Although extended resection achieved more lymph node removal (16 [12, 19] vs. 10 [5, 13.5], *p* < 0.001, Figure 5c), it may also mean more postoperative complications (33.3% vs 11.6%, *p* = 0.012 Figure 5e) and a longer hospital stay (6 [5, 9] days vs. 5 [4, 6] days, *p* = 0.01, Figure 5d). In addition, patients may not benefit more from the surgical strategy of extended resection according to the result of PFS and OS (*p* = 0.52 and 0.85, respectively, Figure 5f,g).

#### Number of lymph nodes removed

**FIGURE 6:**
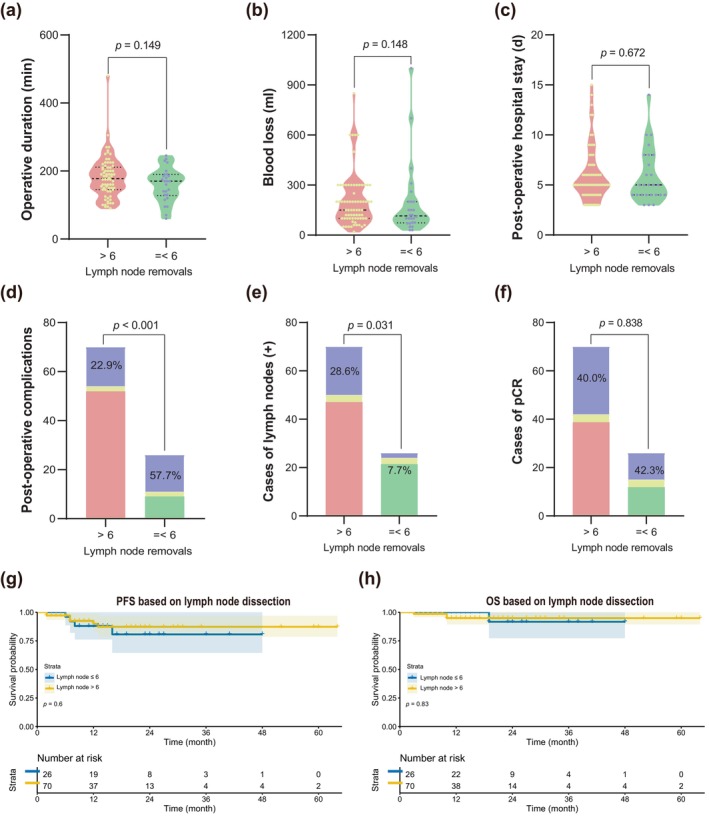
Comparison of postoperative results stratified by lymph node removals. (a) Operative duration (min). (b) Intraoperative blood loss (mL). (c) Postoperative hospital stay (days). (d) Rate of postoperative complications (%). (e) Rate of positive lymph nodes (%). (f) Rate of pCR (%). (g) Kaplan–Meier curves for PFS among two groups. (h) Kaplan–Meier curves for OS among two groups. PFS, progression‐free survival; OS, overall survival.

Regarding the strategy of lymph nodes dissected, we found that the more lymph‐node dissection group had a longer operation time (177.5 [145, 211] min vs. 170 [128, 190] min, *p* = 0.149, Figure 6a) and more intraoperative blood loss (150 [100, 300] mL vs. 115 [74, 200] mL, *p* = 0.148, Figure 6b) than the less lymph‐node dissection group. A high rate of positive lymph nodes was detected intraoperatively (28.6% vs. 7.7%, *p* = 0.031, Figure 6e), and fewer postoperative complications occurred (22.9% vs. 57.7%, *p* < 0.001, Figure 6d) in the more lymph node dissection groups. Interestingly, the rate of pCR was not affected by the number of lymph nodes dissected, and there was no difference in the length of postoperative hospital stay (5 [4, 7] days vs. 5 [4, 8] days, *p* = 0.672, Figure 6c) between the two groups. Furthermore, there was no significant difference in PFS and OS between the less lymph node dissection group and the more lymph node dissection group (*p* = 0.6 and 0.83, respectively, Figure 6g,h). This finding showed that the choice of a relatively aggressive lymph node dissection strategy did not affect the survival and prognosis of patients, and surgeons may consider the appropriate lymph node dissection strategy carefully according to their own experience and our results showed that the choice of a relatively aggressive lymph node dissection strategy did not affect the survival and prognosis of patients, and surgeons may consider the appropriate lymph node dissection strategy carefully according to their own experience and surgical circumstances.

#### Survival analysis in the subgroup

**FIGURE 7:**
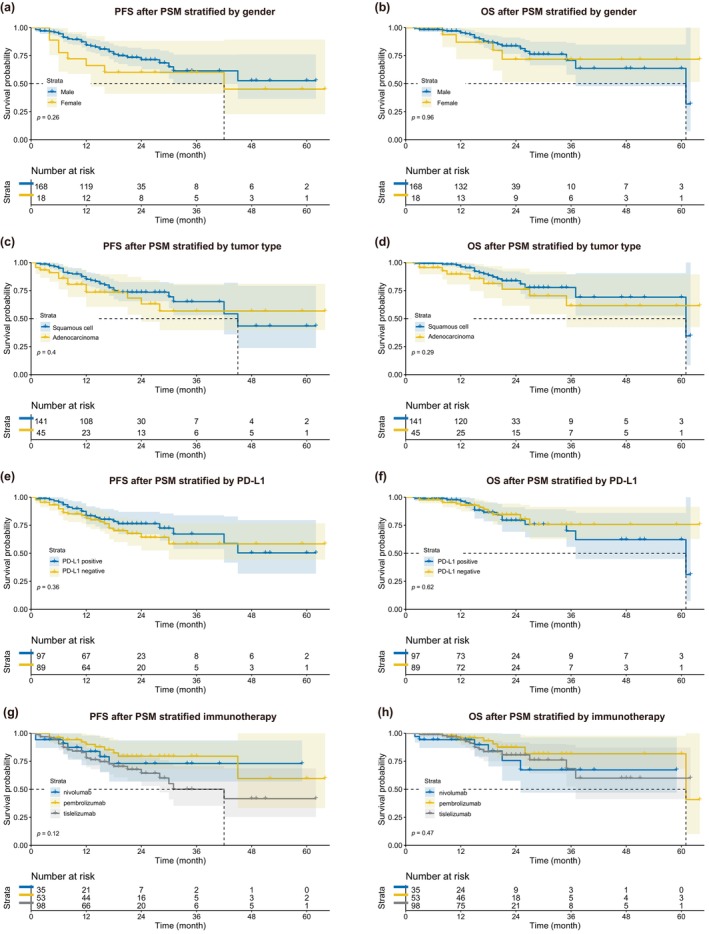
Kaplan–Meier survival curves for each subgroup after propensity score matching. (a) PFS between male and female. (b) OS between male and female. (c) PFS between squamous cell carcinoma and adenocarcinoma. (d) OS between squamous cell carcinoma and adenocarcinoma. (e) PFS between PD‐L1 positive and negative. (f) OS between PD‐L1 positive and negative. (g) PFS stratified by immunotherapy. (h) OS stratified by immunotherapy. PFS, progression‐free survival; OS, overall survival; PSM, propensity score matching; PD‐L1, programmed death‐ligand 1.

In the subgroup analyses, we found no significant differences between the survival curves of different genders (male vs. female), different tumor histological types (squamous cell carcinoma vs. adenocarcinoma), different PD‐L1 expression (positive vs. negative), and different immunotherapy regimens (tislelizumab vs. nivolumab vs. pembrolizumab) (Figure 7).

We further performed univariate and multivariate survival analysis on postoperative patients. We found that R0 resection was a protective factor for PFS of patients with surgery (HR: 0.183, 95% CI: 0.062–0.538, *p* = 0.002) (Table 4).

**TABLE 4:** Analysis of progression‐free survival in surgical group.

Variables	Univariate analysis*					Multivariate analysis				
	B	SE	Wals	OR (95% CI)	*p*‐value*	B	SE	Wals	OR (95% CI)	*p*‐value
Age (≥60 vs. <60 years)	0.664	0.484	1.883	1.942 (0.753–5.011)	0.170					
Gender (female vs. male)	0.875	0.571	2.353	2.400 (0.784–7.345)	0.125					
BMI pre‐nICT (≥22 vs. <22 kg/m^2^)	−0.571	0.475	1.448	0.565 (0.223–1.432)	0.229					
BMI post‐nICT (≥22 vs. <22 kg/m^2^)	−0.065	0.501	0.017	0.937 (0.351–2.502)	0.897					
ΔBMI (increase vs. decrease)	0.323	0.568	0.324	1.381 (0.454–4.205)	0.569					
PNI (>45 vs. ≤45)	−0.683	0.484	1.991	0.505 (0.196–1.304)	0.158					
CCI (>2 vs. ≤2)	−0.198	0.528	0.141	0.820 (0.292–2.307)	0.707					
Smoking status (present vs. absent)	−1.035	0.504	4.224	0.355 (0.132–0.953)	**0.040**	−0.678	0.543	1.562	0.507 (0.175–1.470)	0.211
Alcohol use (yes vs. no)	−0.574	0.506	1.286	0.563 (0.209–1.519)	0.257					
FEV1% (≥80% vs. <80%)	0.907	0.752	1.454	2.476 (0.0.567–10.806)	0.228					
Tumor size post‐nICT (>3 vs. ≤3 cm)	−0.646	0.751	0.740	0.524 (0.120–2.284)	0.390					
Δ tumor size reduction (>2.5 vs. ≤2.5 cm)	0.940	0.526	3.192	2.561 (0.913–7.186)	**0.074**	0.988	0.548	3.255	2.687 (0.918–7.862)	0.071
Tumor location (right vs. left lobe)	0.091	0.152	0.354	1.095 (0.812–1.476)	0.552					
Complete resection (R0 vs. R1 and R2)	−2.304	0.474	23.634	0.100 (0.039–0.253)	**<0.001**	−1.700	0.551	9.525	0.183 (0.062–0.538)	**0.002**
Minimally invasive surgery (yes vs. no)	−0.536	0.500	1.150	0.585 (0.219–1.559)	0.284					
Tumor type (adenocarcinoma vs. squamous)	−0.115	0.526	0.048	0.891 (0.318–2.497)	0.826					
Lymph node (+)	0.436	0.682	0.408	1.546 (0.406–5.885)	0.523					
Lymph node dissection (>6 vs. ≤6)	−0.329	0.628	0.276	0.719 (0.210–2.461)	0.600					
Vascular or bronchial reconstruction	−0.391	0.628	0.389	0.676 (0.198–2.313)	0.533					
pCR	−1.820	0.753	5.837	0.162 (0.037–0.709)	**0.016**	−1.391	0.788	3.113	0.249 (0.053–1.167)	0.078
Adjuvant therapy cycle										
3–4 versus 0–2	1.439	0.690	4.344	4.216 (1.090–16.311)	**0.037**	0.480	0.770	0.390	1.617 (0.358–7.306)	0.533
≥5 versus 0–2	1.231	0.677	3.300	3.424 (0.908–12.916)	**0.069**	0.845	0.732	1.322	2.329 (0.554–9.784)	0.248

*Note*: Bold values indicated less than 0.1 and may be significant values.

Abbreviations: BMI, body mass index; CCI, Charlson comorbidity index; CI, confidence interval; nICT, neoadjuvant immunotherapy combined with chemotherapy; OR, odds ratio: pCR, pathological complete response; PNI, prognostic nutritional index; Δ, change in values before and after neoadjuvant immunotherapy combined with chemotherapy; WALS, weighted‐average least squares.

*, parameters with *p* < 0.1 were included in the multivariate analysis.

## DISCUSSION

The treatment strategies for advanced stage III NSCLC have been a subject of considerable controversy in the field of lung cancer treatment due to the inherent tumor heterogeneity. The decision‐making process concerning surgical or nonsurgical intervention for these patients often depends on the clinicians' experience and subjective judgment in various clinical practice scenarios.^11^ Experienced physicians can make informed decisions about the potential benefits of surgical interventions for advanced stage III lung cancer based on their previous diagnostic and treatment experiences. Conversely, less experienced doctors may opt for more conservative nonsurgical treatment approaches to mitigate the risks associated with the treatment. In light of these considerations, we formulated a hypothesis: Given the challenge in establishing a uniform criterion to determine resectability in advanced stage III NSCLC due to factors such as doctors' subjective experience, medical skill levels, or medical institution /conditions, could we optimize the lung cancer treatment strategy by assessing whether patients can undergo surgery when tumors demonstrate a favorable response after a certain cycle of immunotherapy and chemotherapy, thereby further improving patient prognosis? To address this question, we retrospectively analyzed the survival and prognosis of advanced stage NSCLC who underwent either surgery or nonsurgery after 2–4 cycles of neoadjuvant therapy. We meticulously compared survival outcomes before and after propensity score matching. The cumulative findings from survival analysis, univariate and multivariate analysis strongly indicated that patients with subsequent surgical intervention exhibited a significantly improved prognosis.

The advent of immune checkpoint inhibitors (ICIs) as an essential neoadjuvant option has greatly enhanced the prognosis of patients with resectable lung cancer.^12^ ICIs effectively activate a robust tumor‐specific T cell response systemically, leading to tumor clearance and metastasis inhibition, thereby extending patients' tumor‐free survival and overall survival. Although many experts consider surgical resection to be unnecessary following the PACIFIC immunotherapy pattern,^13^ the potential improvement in patient prognosis with the combination of ICIs and surgery for unresectable advanced stage III NSCLC remains a topic of contention. This also elucidates the subjective and empirical selection challenges faced by different doctors in determining the resectability of advanced stage lung cancer. In recent years, several doctors have found that surgical intervention after immunotherapy for unresectable advanced stage III NSCLC can be a viable strategy to enhance patient prognosis based on clinical practice with small sample sizes. Deng et al. recently reported a case series comprising 31 unresectable stage IIIB NSCLC patients who underwent surgical resection after at least two cycles of PD‐1 blockades plus platinum‐based chemotherapy.^14^ Their study demonstrated a 71.0% downstaging rate and a longer DFS/PFS compared to those without surgery. Liang et al.^10^ also conducted surgical resection for seven patients with locally advanced stage III NSCLC after immunotherapy, all of whom remained alive with no recurrence after nearly a 3‐year follow‐up. Our results not only strongly support their findings but also underscore that regardless of type of surgery, as long as advanced stage III NSCLC patients receive a certain cycle of immunotherapy combined with chemotherapy and tumor achieves favorable response as well as being resectable, surgery represents a superior option for enhancing prognosis.

Furthermore, to optimize the surgical strategy, we conducted a comprehensive analysis and discussion concerning the influence of different surgical strategies on patients' prognosis, focusing on three key aspects: minimally invasive surgery (MIS), the extent of surgical resection, and lymph node resection. Post‐immunotherapy, thoracic anatomy of patients, particularly the hilar and mediastinal regions, can be impacted by ICIs, especially in patients whose tumors respond to the drugs. The tumor cell‐killing effect of ICIs leads to adhesion and fibrosis repair in the local area, posing significant challenges to subsequent surgical planning.^15^ Traditional thoracotomy effectively addresses anatomical structure adhesion and ensures accurate tumor removal after immunosuppressive therapy. However, it also introduces challenges such as significant trauma, increased postoperative complications, and extended recovery periods. MIS has revolutionized surgical treatment for both early and advanced NSCLC owing to its advantages of reduced surgical trauma, fewer postoperative complications, and shorter postoperative recovery times.^16^ While several articles have mentioned the use of MIS in the surgical treatment of lung cancer patients after immunotherapy,^16^, ^17^ reports directly comparing MIS with thoracotomy have been limited. Recently, Zhang et al.^18^ conducted a retrospective study among stage IB–IIIB lung cancer patients and found that VATS achieved comparable efficacy to open surgery, with shorter ICU stays for patients, indicating the safety, efficacy, and feasibility of VATS as a surgical strategy for patients after immunotherapy.^19^ Our results similarly demonstrated that patients who underwent MIS after immunotherapy had equally favorable prognoses to those who underwent thoracotomy. MIS not only maintained a clear field of vision for lymph node dissection but also offered advantages such as shorter operation times, less intraoperative blood loss, and fewer postoperative complications.

Regarding extensive resection in surgical treatment, the aim is to achieve R0 resection, minimizing residual tumor to prevent subsequent recurrence. However, the reduction in extended surgical resection for borderline resectable lung cancer, such as advanced stage III NSCLC, due to the use of ICIs has been a topic of debate.^20^ Current clinical studies that have concluded ICIs can reduce tumor size and potentially obviate the need for extended resection primarily focused on resectable lung cancer. The association between preoperative ICI treatment and reduced resection extent due to tumor shrinkage after treatment remain uncertain for unresectable lung cancer.^21^ In the CheckMate 816 trial, the tumor reduction rate was higher in the preoperative ICI group compared to the group without ICIs. However, in the NADIM II, Neotorch, and KEYNOTE‐671 trials, the proportion of patients with tumor reduction after using ICIs was similar to the no‐ICIs group.^12^, ^22^, ^23^, ^24^, ^25^, ^26^, ^27^

Therefore, it is worth exploring whether appropriate extended resection after immunotherapy is beneficial to the prognosis of patients with advanced stage III NSCLC, provided the patient's tolerance and safety of surgery are carefully evaluated. Our subgroup analysis indicated that OS and PFS were comparable in the lobectomy and extended resection groups. Although more lymph nodes were dissected, the extended resection group exhibited longer operation times, increased intraoperative blood loss, prolonged postoperative hospital stays, and a higher incidence of postoperative complications compared with the lobectomy group. These results suggest that patients with advanced stage III lung cancer who have received immunotherapy do not benefit significantly from extended resection. Surgeons should exercise caution in selecting extended resection procedures. For patients deemed to be at a heightened risk, it is advisable to employ intraoperative frozen pathological analysis to precisely ascertain margin status and thereby mitigate the extent of surgical excision.

Moreover, while immunotherapy has substantially reduced the impact of the tumor, extended lymph node dissection during surgery after immunotherapy can effectively identify potential metastatic lymph nodes and reduce the risk of postoperative recurrence. Additionally, extended lymph node dissection provides more accurate postoperative pathological staging, offering vital insights for subsequent treatment and prognosis evaluation. For locally advanced lung cancer, the NCCN guidelines continue to generate controversy regarding whether extended lymph node dissection should be performed.^28^ However, in recent years, numerous studies have suggested that extended lymph node dissection after immunotherapy is crucial for improving patient prognosis. Corsini et al. found that patients with ypN1‐2 were at risk of early recurrence, regardless of whether the primary tumor achieved major pathological response (MPR) in response to immunotherapy.^29^ Studies on other malignant tumors such as esophageal and gastric cancer have also demonstrated that the number of metastatic lymph nodes can better evaluate and predict prognosis.^30^, ^31^ The study by He et al. similarly suggested that intraoperative detection of 16 lymph nodes was a significant marker for better prognosis in patients.^32^

Therefore, it is imperative to perform more lymph node dissection during the operation. Our results showed that although there was no significant difference in OS and PFS between the less lymph node dissection group (≤6) and the more lymph node dissection group (>6), extended lymph node dissection did not significantly affect operation time, intraoperative blood loss, pCR rate, or postoperative hospital stay. While the advantage of extended lymph node dissection in improving the survival prognosis of patients may have been impacted by the short follow‐up time for some patients, our results revealed that a higher number of lymph nodes removed increased the probability of detecting positive lymph nodes, facilitating surgical strategy adjustments during and after surgery to further eliminate tumors. Intriguingly, the incidence of postoperative complications in patients undergoing extended lymph node dissection was lower than that in the limited lymph node dissection group. This could be attributed to the following reasons. First, cases with a higher potential for complications often necessitate a more intricate tumor resection, and the surgeon may opt to minimize the removal of lymph nodes. Second, sample bias in this study. Third, additional factors may also contribute, warranting further investigation. In summary, our findings strongly advocate for the implementation of lymph node dissection, particularly targeting nodes with potential positivity. This procedure should be carried out whenever feasible, leveraging the surgeon's proficient surgical expertise and the relative surgical tractability.

Immunotherapy is profoundly altering the approach to treating lung cancer, presenting a promising outlook. In this era of immunotherapeutic advancements, the following personal reflections are offered. First, strengthening the collaboration among multidisciplinary teams (MDT) is imperative to circumvent an indiscriminate approach to lung cancer. The occurrence and development of lung cancer exhibit significant heterogeneity that has yet to be fully comprehended. Second, conducting surgery for advanced‐stage lung cancer poses considerable challenges. Neoadjuvant therapies, including immunotherapy, may amplify fibrous tissue proliferation and disturb anatomical structures within the surgical area. Consequently, despite the prevailing use of VATS today, there remains a pressing necessity to augment surgical expertise. Third, post‐immunotherapy, we have observed numerous cases achieving a pathological complete response (pCR). This raises the question of whether surgery is necessary in these cases. Moreover, in instances of achieving a major pathological response (MPR), what the appropriate extent of surgical intervention should be remains the subject of intense debate. The present study offers an initial exploration, and it is anticipated that forthcoming research will delve more deeply into this matter.

However, the present study had several limitations. First, the follow‐up duration for certain patients included in the study was insufficient, resulting in truncated values that may impact the accuracy of survival analysis within the two groups. Second, given the retrospective nature of this study, notwithstanding our diligent implementation of propensity score matching for essential clinical indicators, we acknowledge the possibility of patient selection bias across various departments. Lastly, the sample size in this study was small. We aspire to conduct future research with a more expansive sample size and comprehensive follow‐up data to enhance the robustness of our findings and conclusions.

In conclusion, our findings suggest that patients with advanced stage III NSCLC, whose tumors achieved favorable response following immunotherapy and subsequent surgical intervention, exhibited a more favorable prognosis compared to those who maintained immunotherapy alone. Moreover, within this subgroup of surgically treated patients, VATS proved to be a suitable surgical approach in this complex situation. Notably, the intraoperative selection between extended lobectomy and a more aggressive lymph node dissection strategy did not significantly influence patient survival or disease recurrence. Clinicians can make informed decisions based on their expertise and the patient's condition during the surgical process. Despite encountering formidable obstacles, such as protracted surgical procedures and associated trauma, we must rise to the challenge and unleash the power of surgery after immunotherapy in advanced NSCLC.

## AUTHOR CONTRIBUTIONS

Conception and design: Fuqiang Dai, Lunxu Liu, Bo Deng, and Yun Wang. Administrative support: Lunxu Liu, Yun Wang, and Tao Li. Provision of study materials or patients: Fuqiang Dai, Bo Deng, Xintian Wang, and Longyong Mei. Collection and assembly of data: Fuqiang Dai, Cong Chen, Guanyu Zhou, Nanzhi Luo, and Wenjing Zhou. Data analysis and interpretation: Fuqiang Dai and Guanyu Zhou. Manuscript writing: All authors. Final approval of manuscript: All authors.

## CONFLICT OF INTEREST STATEMENT

No conflict of interest exits in the submission of this manuscript, and manuscript is approved by all authors for publication.

## References

[1] Siegel RL , Miller KD , Wagle NS , Jemal A . Cancer statistics, 2023. CA Cancer J Clin. 2023;73(1):17–48. 36633525 10.3322/caac.21763

[2] Remon J , Soria JC , Peters S . Early and locally advanced non‐small‐cell lung cancer: an update of the ESMO clinical practice guidelines focusing on diagnosis, staging, systemic and local therapy. Ann Oncol. 2021;32(12):1637–1642. 34481037 10.1016/j.annonc.2021.08.1994

[3] Temel JS , Petrillo LA , Greer JA . Patient‐centered palliative care for patients with advanced lung cancer. J Clin Oncol. 2022;40(6):626–634. 34985932 10.1200/JCO.21.01710

[4] Ettinger DS , Wood DE , Aisner DL , Akerley W , Bauman JR , Bharat A , et al. NCCN guidelines® insights: non‐small cell lung cancer, version 2.2023. J Natl Compr Canc Netw. 2023;21(4):340–350. 37015337 10.6004/jnccn.2023.0020

[5] Chaft JE , Oezkan F , Kris MG , Bunn PA , Wistuba II , Kwiatkowski DJ , et al. Neoadjuvant atezolizumab for resectable non‐small cell lung cancer: an open‐label, single‐arm phase II trial. Nat Med. 2022;28(10):2155–2161. 36097216 10.1038/s41591-022-01962-5 PMC9556329

[6] Ulas EB , Dickhoff C , Schneiders FL , Senan S , Bahce I . Neoadjuvant immune checkpoint inhibitors in resectable non‐small‐cell lung cancer: a systematic review. ESMO Open. 2021;6(5):100244. 34479033 10.1016/j.esmoop.2021.100244 PMC8414043

[7] Akinboro O , Drezner N , Amatya A , Runyan J , Fourie‐Zirkelbach J , Zhao M , et al. US Food and Drug Administration approval summary: nivolumab plus platinum‐doublet chemotherapy for the neoadjuvant treatment of patients with Resectable non‐small‐cell lung cancer. J Clin Oncol. 2023;41(17):3249–3259. 37141544 10.1200/JCO.22.02509 PMC10256356

[8] Reck M , Rabe KF . Precision diagnosis and treatment for advanced non‐small‐cell lung cancer. N Engl J Med. 2017;377(9):849–861. 28854088 10.1056/NEJMra1703413

[9] Liang H , Yang C , Gonzalez‐Rivas D , Zhong Y , He P , Deng H , et al. Sleeve lobectomy after neoadjuvant chemoimmunotherapy/chemotherapy for local advanced non‐small cell lung cancer. Transl Lung Cancer Res. 2021;10(1):143–155. 33569300 10.21037/tlcr-20-778 PMC7867787

[10] Bott MJ , Cools‐Lartigue J , Tan KS , Dycoco J , Bains MS , Downey RJ , et al. Safety and feasibility of lung resection after immunotherapy for metastatic or unresectable tumors. Ann Thorac Surg. 2018;106(1):178–183. 29550207 10.1016/j.athoracsur.2018.02.030 PMC6357770

[11] Daly ME , Singh N , Ismaila N , Antonoff MB , Arenberg DA , Bradley J , et al. Management of Stage III non‐small‐cell lung cancer: ASCO guideline. J Clin Oncol. 2022;40(12):1356–1384. 34936470 10.1200/JCO.21.02528

[12] Provencio M , Nadal E , Insa A , García‐Campelo MR , Casal‐Rubio J , Dómine M , et al. Neoadjuvant chemotherapy and nivolumab in resectable non‐small‐cell lung cancer (NADIM): an open‐label, multicentre, single‐arm, phase 2 trial. Lancet Oncol. 2020;21(11):1413–1422. 32979984 10.1016/S1470-2045(20)30453-8

[13] Naidoo J , Antonia S , Wu YL , Cho BC , Thiyagarajah P , Mann H , et al. Brief report: durvalumab after chemoradiotherapy in unresectable stage III EGFR‐mutant NSCLC: a post hoc subgroup analysis from PACIFIC. J Thorac Oncol. 2023;18(5):657–663. 36841540 10.1016/j.jtho.2023.02.009

[14] Deng H , Liu J , Cai X , Chen J , Rocco G , Petersen RH , et al. Radical minimally invasive surgery after Immuno‐chemotherapy in initially‐unresectable stage IIIB non‐small cell lung cancer. Ann Surg. 2022;275(3):e600–e602. 34596079 10.1097/SLA.0000000000005233

[15] Bott MJ , Yang SC , Park BJ , Adusumilli PS , Rusch VW , Isbell JM , et al. Initial results of pulmonary resection after neoadjuvant nivolumab in patients with resectable non‐small cell lung cancer. J Thorac Cardiovasc Surg. 2019;158(1):269–276. 30718052 10.1016/j.jtcvs.2018.11.124 PMC6653596

[16] Cao C , Guo A , Chen C , Chakos A , Bott M , Yang CJ , et al. Systematic review of neoadjuvant immunotherapy for patients with non‐small cell lung cancer. Semin Thorac Cardiovasc Surg. 2021;33(3):850–857. 33444765 10.1053/j.semtcvs.2020.12.012 PMC8273189

[17] Leal TA , Ramalingam SS . Neoadjuvant therapy gains FDA approval in non‐small cell lung cancer. Cell Rep Med. 2022;3(7):100691. 35858590 10.1016/j.xcrm.2022.100691 PMC9381414

[18] Zhang B , Xiao Q , Xiao H , Wu J , Yang D , Tang J , et al. Perioperative outcomes of video‐assisted thoracoscopic surgery versus open thoracotomy after neoadjuvant chemoimmunotherapy in Resectable NSCLC. Front Oncol. 2022;12:858189. 35712494 10.3389/fonc.2022.858189 PMC9194512

[19] Dai F , Wu X , Wang X , Li K , Wang Y , Shen C , et al. Neoadjuvant immunotherapy combined with chemotherapy significantly improved patients' overall survival when compared with neoadjuvant chemotherapy in non‐small cell lung cancer: a cohort study. Front Oncol. 2022;12:1022123. 36353552 10.3389/fonc.2022.1022123 PMC9637677

[20] Provencio M , Calvo V , Romero A , Spicer JD , Cruz‐Bermúdez A . Treatment sequencing in Resectable lung cancer: the good and the bad of adjuvant versus neoadjuvant therapy. Am Soc Clin Oncol Educ Book. 2022;42:1–18. 10.1200/EDBK_358995 35561296

[21] Mountzios G , Remon J , Hendriks LEL , García‐Campelo R , Rolfo C , Van Schil P , et al. Immune‐checkpoint inhibition for resectable non‐small‐cell lung cancer ‐ opportunities and challenges. Nat Rev Clin Oncol. 2023;20:664–677. 37488229 10.1038/s41571-023-00794-7

[22] Wakelee H , Liberman M , Kato T , Tsuboi M , Lee SH , Gao S , et al. Perioperative pembrolizumab for early‐stage non‐small‐cell lung cancer. N Engl J Med. 2023;389(6):491–503. 37272513 10.1056/NEJMoa2302983 PMC11074923

[23] Forde PM , Spicer J , Lu S , Provencio M , Mitsudomi T , Awad MM , et al. Neoadjuvant nivolumab plus chemotherapy in Resectable lung cancer. N Engl J Med. 2022;386(21):1973–1985. 35403841 10.1056/NEJMoa2202170 PMC9844511

[24] Provencio M , Nadal E , González‐Larriba JL , Martínez‐Martí A , Bernabé R , Bosch‐Barrera J , et al. Perioperative nivolumab and chemotherapy in stage III non‐small‐cell lung cancer. N Engl J Med. 2023;389(6):504–513. 37379158 10.1056/NEJMoa2215530

[25] Provencio M , Serna‐Blasco R , Nadal E , Insa A , García‐Campelo MR , Casal Rubio J , et al. Overall survival and biomarker analysis of neoadjuvant nivolumab plus chemotherapy in operable stage IIIA non‐small‐cell lung cancer (NADIM phase II trial). J Clin Oncol. 2022;40(25):2924–2933. 35576508 10.1200/JCO.21.02660 PMC9426809

[26] Heymach JV , Harpole D , Mitsudomi T , Taube JM , Galffy G , Hochmair M , et al. Abstract CT005: AEGEAN: a phase 3 trial of neoadjuvant durvalumab + chemotherapy followed by adjuvant durvalumab in patients with resectable NSCLC. Cancer Res. 2023;83(8_Supplement):CT005.

[27] Lu S , Wu L , Zhang W , Zhang P , Wang W , Fang W , et al. Perioperative toripalimab + platinum‐doublet chemotherapy vs chemotherapy in resectable stage II/III non‐small cell lung cancer (NSCLC): interim event‐free survival (EFS) analysis of the phase III NEOTORCH study. J Clin Oncol. 2023;41(16_suppl):8501.

[28] Ettinger DS , Wood DE , Aisner DL , Akerley W , Bauman JR , Bharat A , et al. NCCN guidelines insights: non‐small cell lung cancer, version 2.2021. J Natl Compr Canc Netw. 2021;19(3):254–266. 33668021 10.6004/jnccn.2021.0013

[29] Corsini EM , Weissferdt A , Pataer A , Zhou N , Antonoff MB , Hofstetter WL , et al. Pathological nodal disease defines survival outcomes in patients with lung cancer with tumour major pathological response following neoadjuvant chemotherapy. Eur J Cardiothorac Surg. 2021;59(1):100–108. 32864702 10.1093/ejcts/ezaa290

[30] Zhang CH , Li YY , Zhang QW , Biondi A , Fico V , Persiani R , et al. The prognostic impact of the metastatic lymph nodes ratio in colorectal cancer. Front Oncol. 2018;8:628. 30619762 10.3389/fonc.2018.00628 PMC6305371

[31] Rice TW , Ishwaran H , Ferguson MK , Blackstone EH , Goldstraw P . Cancer of the esophagus and esophagogastric junction: an eighth edition staging primer. J Thorac Oncol. 2017;12(1):36–42. 27810391 10.1016/j.jtho.2016.10.016 PMC5591443

[32] Liang W , He J , Shen Y , Shen J , He Q , Zhang J , et al. Impact of examined lymph node count on precise staging and long‐term survival of resected non‐small‐cell lung cancer: a population study of the US SEER database and a Chinese multi‐institutional registry. J Clin Oncol. 2017;35(11):1162–1170. 28029318 10.1200/JCO.2016.67.5140 PMC5455598

